# Emergence of multidrug-resistant ST876 serotype 14 *Streptococcus pneumoniae* with spontaneous mutations driving pathogenesis: genomic and clinical insights from pneumococcal pericarditis and global screening

**DOI:** 10.3389/fcimb.2026.1723478

**Published:** 2026-03-24

**Authors:** Lin Liu, Shouyuan Chen, Tingting Hong, Ying Fu, Yining Ye, Yuxiaoting Li, Tingting Li, Tailong Lei, Yan Chen, Yanfei Wang, Dongdong Zhao, Xueqing Wu, Yunsong Yu

**Affiliations:** 1 Laboratory Medicine Center, Department of Clinical Laboratory, Zhejiang Provincial People’s Hospital, Affiliated People’s Hospital, Hangzhou Medical College, Hangzhou, Zhejiang, China; 2 Department of Infectious Disease, Sir Run Run Shaw Hospital, Zhejiang University School of Medicine, Hangzhou, Zhejiang, China; 3 Department of Clinical Laboratory, Sir Run Run Shaw Hospital, School of Medicine, Zhejiang University, Hangzhou, Zhejiang, China; 4 Zhejiang Provincial Engineering Research Center of Innovative Instruments for Precise Pathogen Detection, Hangzhou, Zhejiang, China; 5 Key Laboratory of Microbial Technology and Bioinformatics of Zhejiang Province, Hangzhou, Zhejiang, China; 6 Regional Medical Center for National Institute of Respiratory Diseases, Sir Run Run Shaw Hospital, Zhejiang University School of Medicine, Hangzhou, Zhejiang, China; 7 Department of Clinical Laboratory, the First People’s Hospital of Linhai, Linhai, Zhejiang, China; 8 Department of Clinical Laboratory, Jiangshan People’s Hospital, Quzhou, Zhejiang, China

**Keywords:** CiaH, FabT, heart infection, pericarditis, pneumococcus, serotype 14, ST876

## Abstract

**Background:**

*Streptococcus pneumoniae* pericarditis is rare and lethal, yet how the pathogen evolves within the host during blood-to-pericardium spread remains unknown.

**Methods:**

Two ST876 serotype 14 strains, hz23B (blood) and hz23P (pericardial effusion), were isolated from one purulent pericarditis patient. We determined antimicrobial susceptibility test (AST) by broth microdilution, performed whole-genome sequencing and mutation detection, multi-locus sequencing typing, and phylogenetic analysis against 1429 global serotype 14 genomes, modeled protein structures for unique mutations, and quantified growth under anaerobic/low-nutrient conditions, plus adherence and invasion on nasopharyngeal and lung epithelial cell lines.

**Results:**

Both isolates showed an identical AST profile, where a multidrug-resistant phenotype was observed. Genomes differed by only 36 SNPs, while hz23P acquired CiaH_W192L (transmembrane kinase loss) and FabT_E21A (DNA-binding reduction). These changes conferred hz23P’s enhanced growth in THY and BSA+G under anaerobic stress (*p* < 0.0001) but significantly reduced adherence (*p* < 0.0001) and invasiveness (*p* < 0.01) compared with hz23B. Global screening identified ST876 as a unique clone that carries pericarditis-linked mutations and successfully dominated China due to multidrug resistance.

**Conclusions:**

We provide the first real-time evidence that ST876 serotype 14 pneumococci evolve within the host to survive the pericardial environment by attenuating virulence and energy. The global screen underscores the urgent need for surveillance targeting this multidrug-resistant and virulent clone.

## Introduction

Invasive pneumococcal disease (IPD) poses a significant and severe threat, often leading to high morbidity and mortality rates, such as bacteremia, meningitis, and cardiac infections (Narciso et al., 2025). The impact of pneumococcus on the respiratory or central nervous systems has been extensively studied (Kadioglu et al., 2008; Gil et al., 2023), but its effects on cardiac infections, particularly purulent pericarditis, have received relatively little attention. Despite its high mortality, the mechanisms of pneumococcal pericarditis remain poorly understood, with only sporadic case reports available (Cillóniz et al., 2015; Patel et al., 2015; Houlihan et al., 2021). Further research is urgently needed to fully understand the pathogenesis of pneumococcus during heart infections.

Serotypes 3 and 9N are the common strains for pneumococcal pericarditis (Africano et al., 2021). As far as we know, only one case of pericarditis caused by *S. pneumoniae* serotype 14 strain was reported in 2006 in Sweden without further discussion (Kan et al., 2006). We previously reported that the sequence type (ST) 876, serotype 14 strains of *S. pneumoniae* in China present unique characteristics, including high invasiveness, low susceptibility to penicillin, and a special genetic background leading to poor vaccine response (Lan et al., 2023; Liu et al., 2023). These characteristics may contribute to its capability to cause severe cardiovascular complications.

Herein, we first identified two ST876 serotype 14 strains from one patient who suffered from bacteremia and developed purulent pericarditis, isolated from blood and pericardial effusion, respectively. Whole genome analysis, protein structure prediction, and *in vitro* virulence and survival tests were conducted to investigate their pathogenesis. Further global genome screening was utilized to identify resistance and pericarditis virulence of ST876 serotype 14 strains.

## Methods

### Bacterial identification and serotyping

Bacterial cultures were applied to patient samples of peripheral blood and pericardial effusion, and two strains were isolated, respectively, which were designated as hz23B and hz23P. Species identification was performed using mass spectrometry and the optochin test. Latex agglutination (Immulex™ pneumococcus kits) and Quellung reaction were carried out for the serotyping. Next-generation sequencing (NGS) via the Illumina platform of chromosome DNA from both strains was also carried out for species confirmation via rMLST, in *silico* serotyping using SeroBA (v1.0.1) (Epping et al., 2018), and further genome analysis.

### Antimicrobial susceptibility testing

The broth microdilution assay was utilized to determine the minimal inhibitory concentrations (MICs) of several antimicrobial agents, including penicillin (PEN), ceftriaxone (CRO), cefotaxime (CTX), meropenem (MEM), linezolid (LZD), and erythromycin (ERY) against hz23B and hz23P, according to the Clinical and Laboratory Standard Institute (CLSI) protocols. *S. pneumoniae* ATCC 49619 was used as a quality control strain. MIC results were defined according to the 2025 EUCAST (Version 15) guidelines.

### Whole genome analysis

The genomic DNA of all tested strains was extracted using the QIAamp^®^ DNA Mini Kit (Qiagen Valencia, CA) and submitted to sequencing via an Illumina HiSeq X10 platform (Illumina, San Diego, CA). Raw next-generation sequencing (NGS) data were subjected to quality control using fastp v0.23.4 (Chen, 2023) with default parameters for adapter trimming, low-quality read filtering, and base correction. The serotyping results of hz23B and hz23P reported serotype 14 for both strains. To determine whether hz23P and hz23B are the same strain, we conducted a single-nucleotide polymorphism (SNPs) and cgMLST (core genome MLST) analysis between all blood-isolated serotype 14 strains (n=10) in our lab, including hz23B and hz23P, using NGS reads via Snippy (Seemann, [[NoYear]]a) and BacWGSTdb 2.0 (Feng et al., 2021), respectively. More than 20 SNPs will be determined as different strains. Then, the breseq v0.39.0 (Deatherage and Barrick, 2014) was utilized to find the mutation between hz23B and hz23P to further elucidate why strain hz23P caused pericarditis rather than hz23B from a genetic aspect. We also conducted whole-genome analysis against all serotype 14 strains locally (n=63) and globally (n=1429, downloaded from https://pathogen.watch/). For this, the Illumina reads of all strains were assembled by end pairing method using Shovill (Seemann, [[NoYear]]b) (with a minimum splicing length of 200 bp and a minimum coverage of 10-fold. The assemblies were then inputted in multilocus sequence typing (MLST) via PubMLST (https://pubmlst.org). The phylogenetic tree was constructed via PopPUNK 2.7.0 (Lees et al., 2019) and displayed in iTol 7.1 (https://itol.embl.de/) and Microreact (http://microreact.org). BacWGSTdb 2.0 was also used to determine the clonal relationship among these isolates according to the pairwise comparison of the cgMLST alleles and SNP differences.

### Protein structure modeling

As the point mutations in CiaH and FabT are uniquely present in the pericarditis serotype 14 strain hz23P, we conducted the structure modeling for both proteins, CiaH and FabT. The protein sequence of CiaH was first annotated by InterPro (Blum et al., 2025), revealing the presence of an *N*-terminal cytoplasmic rabaptin-like region, two transmembrane helices, and a *C*-terminal WalK-like region with a HisKA-HATPase_c domain architecture. The HisKA domain, also named the dimerization and phosphotransfer (DHp) domain, harbors the phosphorylatable residue H226 within an H-box consensus motif (Stock et al., 1989). The HATPase_c domain, alternatively termed ATP-binding catalytic (CA) domain, contains the conserved N-G1-F-G2-G3 box motifs (Kim and Forst, 2001). Sequence alignment of CiaH from strain hz23P was conducted, and the crystal structure of sensor histidine kinase SrrB in *Staphylococcus aureus* (PDB entry: 6PAJ, chain A) and human RABEP1 (PDB entry:1UT3, chain G) was selected as the templates. After chimera homology modeling, the protein structure was validated and minimized. The CiaH variant of strain hz23B was mutated to W192L from the model with energy minimization and color-coded according to their distinct domains. After sequence alignment, FabT from strain hz23B was found to identify 100% with an X-ray crystal structure in complex with DNA (PDB ID: 6JBX). The FabT variant of strain hz23P was mutated to E21A from the model and minimized.

### Human epithelial cell adhesion, permeability, and translocation test

Since hz23B and hz23P were isolated from different human sites, we would like to compare their pathogenesis, including adhesion, permeability, and translocation. For the adhesion test, we cultured human nasopharyngeal epithelial cells (Detroit 562) and human bronchial epithelial (HBE) cells in a 6-well plate, respectively, following the regular cell culture protocol using a DMEM medium with 10% FBS. When the cells were researched 100% confluent, the same density (10^6^ CFU/mL) of both pneumococcal strains was inoculated on each cell substrate and cultured at 37 °C with 5% CO_2_. At a predetermined time, we washed the wells with PBS three times, collected the bacteria adhering to the cells, and counted the number of bacteria for each well. In the translocation test, the epithelial barrier models were constructed using Detroit 562 and HBE cells in a transwell system with a pore size of 8.0 µm (Corning, USA), which allows bacteria to pass through. When the cells were researched 100% confluent, both pneumococcal strains were inoculated on the cell barriers at a density of 10^6^ CFU/mL and cultured at 37 °C with 5% CO_2_, respectively. At each time point, we collected and counted bacteria from the downside of the Transwells that contained bacteria that crossed the epithelial barrier. We also tested the cell monolayer integrity using the Evans Blue-BSA agent at the end of the translocation culture (8 hours). Briefly, 500 µL 4% BSA was added to the lower chamber, and 100 µL 2 g/L Evans Blue-BSA was added to the upper chamber of each transwell, incubated at 37 °C, 5% CO_2_ for one hour, and the OD was measured at 620nm for the lower champers to evaluate the cell monolayer permeability.

### *S. pneumoniae* point-mutation strains construction

We employed an *rpsL*-based positive-negative selection system to perform markerless gene editing in the hz23B strain. First, the wild-type strain was modified to be streptomycin-resistant (rpsL^+^) to serve as a recipient. Subsequently, Janus selection cassettes targeting the ciaH and fabT genes were constructed via SOE-PCR (structure: upstream homologous arm-chloramphenicol or spectinomycin resistance gene-*rpsL*-downstream homologous arm). The cassettes were introduced into the recipient strain through homologous recombination, and positive selection was performed using the corresponding antibiotics (chloramphenicol at 4 µg/mL or spectinomycin at 200 µg/mL) to obtain mutant intermediates. Next, homologous recombination fragments containing the desired point mutations were transformed into the intermediates. Negative selection was carried out on streptomycin (200 µg/mL) plates to drive a second homologous recombination event, thereby replacing the selection cassette with the target mutant fragment. Finally, PCR and Sanger sequencing confirmed the successful generation of strains with the specific point mutations *ciaH*_W192L or *fabT*_E21A.

### Survival test in the anaerobic and low-nutrient medium

To evaluate the growth advantages of strains hz23B, hz23P, hz23B::*ciaH*_W192L, and hz23B::*fabT*_E21A under distinct nutritional conditions, we utilized nutrient-rich Todd-Hewitt yeast broth (THY) and nutrient-depleted broth: phosphate-buffered saline containing 5 g/L bovine serum albumin supplemented with 1 g/L (BSA+G1g) or 2 g/L (BSA+G2g) glucose. Strains were initially cultured on 5% blood agar plates (37 °C, 5% CO_2_) overnight; then the bacteria were harvested and resuspended in sterile PBS, adjusted to an optical density of OD600 = 0.1. The suspensions were inoculated into fresh broth at a 1:10 ratio, transferred into an anaerobic jar, and incubated statically in a 37 °C incubator with 5% CO_2_ for 4 h, 8 h, and 16 h. At each time point, cultures were serially diluted, and viable bacterial concentrations were determined via plate colony enumeration to assess growth capacity.

### Statistical analysis

The unpaired nonparametric Mann-Whitney test was utilized to evaluate the difference in the bacterial density and Evans blue concentrations between the tested groups. The above statistical analyses were conducted using GraphPad Prism V9.3.1. Other than the indication, the significance was determined with a *p* < 0.001.

## Results

### Strain identification and antimicrobial susceptibility

**Figure 1:**
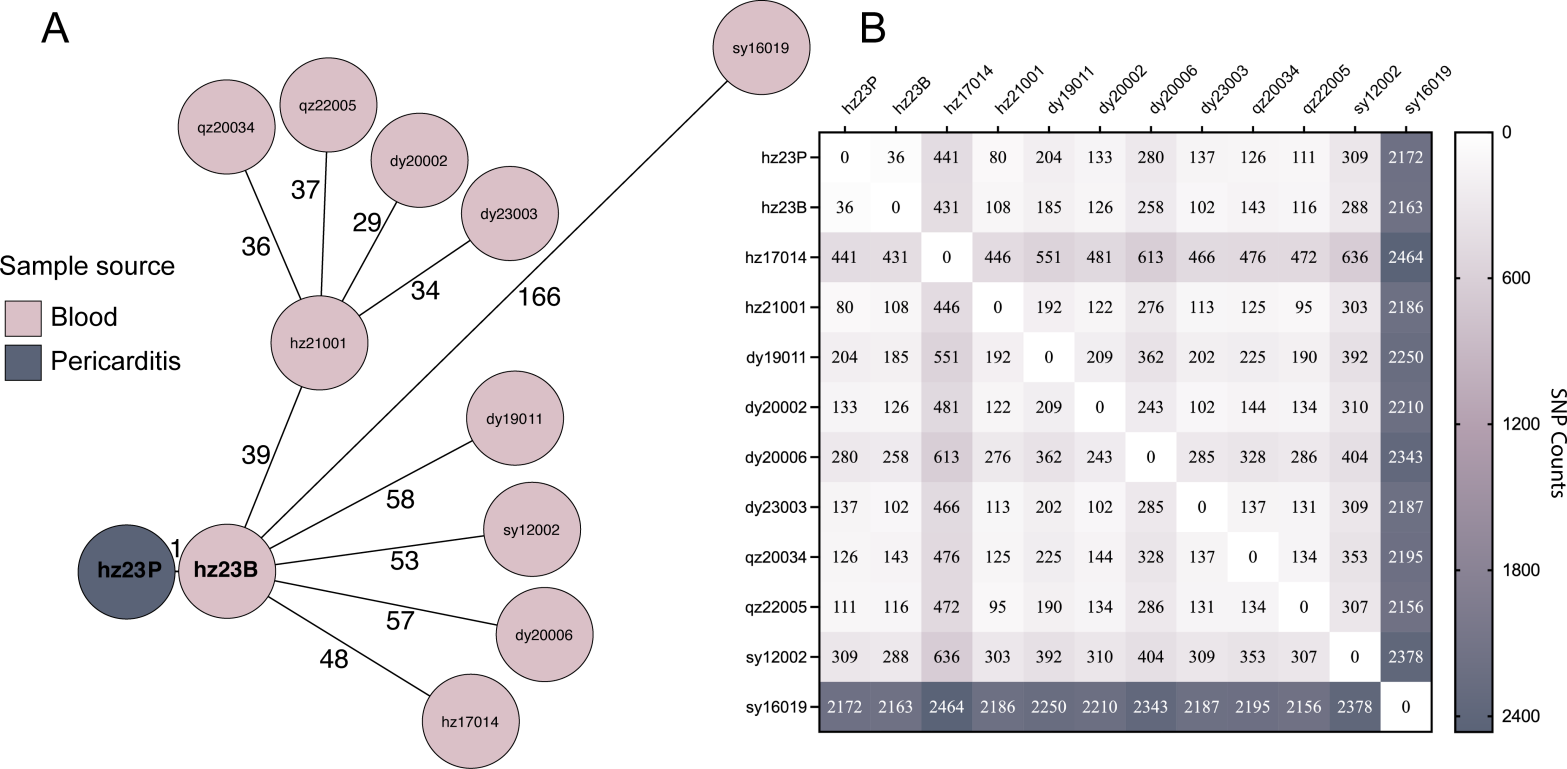
The phylogenetic relationship between hz23B and hz23P and bloodstream infection-associated Serotype 14 *Streptococcus pneumoniae*. **(A)** cgMLST results of hz23B, hz23P, and all other blood-isolated serotype 14 pneumococcal strains (n=10) in our lab. The lines connecting the circles represent the clonal relationships between different isolates, with the numbers on the lines indicating the number of core gene differences. **(B)** The heat map shows the number of single-nucleotide polymorphisms (SNPs) between hz23B, hz23P, and all other blood-isolated serotype 14 pneumococcal strains in our lab.

Two *S. pneumoniae* strains were isolated from one patient’s blood (hz23B) and pericarditis pus (hz23P) after three days of his administration in a tertiary hospital in Hangzhou, Zhejiang Province, China. Via quellung and *in silico* serotyping, we found that hz23B and hz23P are both serotype 14 strains. The two strains are highly similar in genetic background, as they differ by only one core gene (**Figure 1A**). However, they are not the same strain, since they differ by 36 SNPs (**Figure 1B**). Hence, we confirm that hz23B and hz23P are two closely related but distinct *S. pneumoniae* serotype 14 strains from a patient with bacteremia who developed purulent pericarditis. For the antimicrobial susceptibility, as shown in **Table 1**, hz23B and hz23P present the same susceptibility against all tested antimicrobial agents, being sensitive to LZD (MIC = 0.5 μg/mL), resistant to ERY (MIC = 128 μg/mL); sensitive (MIC = 0.5 μg/mL) to MEM, not resistant to CRO (MIC = 1μg/mL) and CTX (MIC = 1μg/mL), and resistant to PEN (MIC = 2 μg/mL) according to the non-endocarditis and non-meningitis break points. According to the endocarditis and meningitis breakpoints, the two strains are resistant to PEN, CRO, CTX, and MEM.

**Table 1:** Antibiotic susceptibility of S. pneumoniae isolates.

MIC (μg/mL)						
NEM/EM Breakpoints	PEN	CRO	CTX	MEM^a^	LZD^b^	ERY^b^
S	≤0.06/≤0.06	≤0.5/≤0.5	≤0.5/≤0.5	≤2/≤0.25	≤2	≤0.25
R	>1/>0.06	>2/>0.5	>2/>0.5	>2/>0.25	>2	>0.25
Strain						
hz23P	2	1	1	0.5	0.5	128
hz23B	2	1	1	0.5	0.5	128
Interpretation NEM/EM						
hz23P	R/R	NS/R	NS/R	S/R	S	R
hz23B	R/R	NS/R	NS/R	S/R	S	R

MIC, Minimum inhibitory concentration; EUCAST breakpoints for NEM, EM: Non-Endocarditis and Non-Meningitis/Endocarditis and Meningitis breakpoints; NA, Not Available; PEN, Penicillin; MEM, Meropenem; CRO, Ceftriaxone; CTX, Cefotaxime; LZD, Linezolid; ERY, Erythromycin. S, Sensitive; R, Resistant; NS, Not Susceptible; “a”, No endocarditis breakpoint, indicates only the meningitis breakpoint; “b”, No differentiation between non-endocarditis/meningitis and endocarditis/meningitis breakpoints.

### Whole-genome analysis

**Figure 2:**
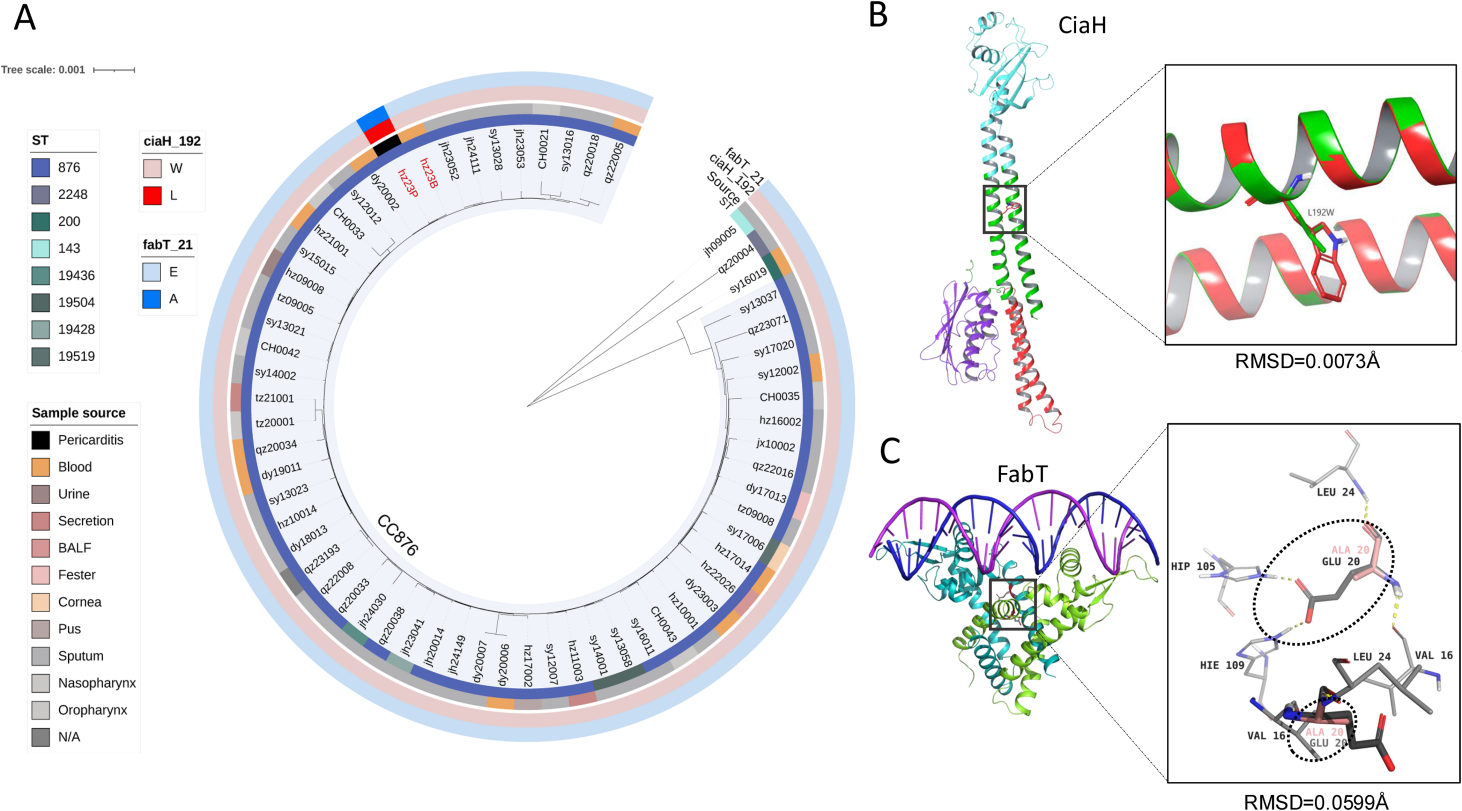
The mutation screening of all serotype 14 *S. pneumoniae* and protein structure prediction and modeling. **(A)** The phylogenetic tree shows the majority of all tested serotype 14 pneumococci (n=63) belonging to CC876, including ST876 and 4 newly assigned STs (19436, 19504, 19428, and 19519) as marked in the inner cycle of the figure. Only one strain (hz23P) was isolated from the pericardial effusion, which was marked as black on the second cycle. The mutations of CiaH_W192L and FabT_E21A were screened for all strains, and only hz23P carries those marked in the outside two cycles. **(B, C)** Protein structure prediction and modeling. Ribbon representations of the CiaH and FabT structure. The mutated hz23P (CiaH_W192L) was marked green in the zoomed-in panel. FabT from strain hz23B (gray in the roomed-in panel) was found to identify 100% with an X-ray crystal structure in a complex with DNA (PDB ID: 6JBX). The FabT variant of strain hz23P carrying FabT_E21A was marked in pink in the zoomed-in panel.

**Figure 3:**
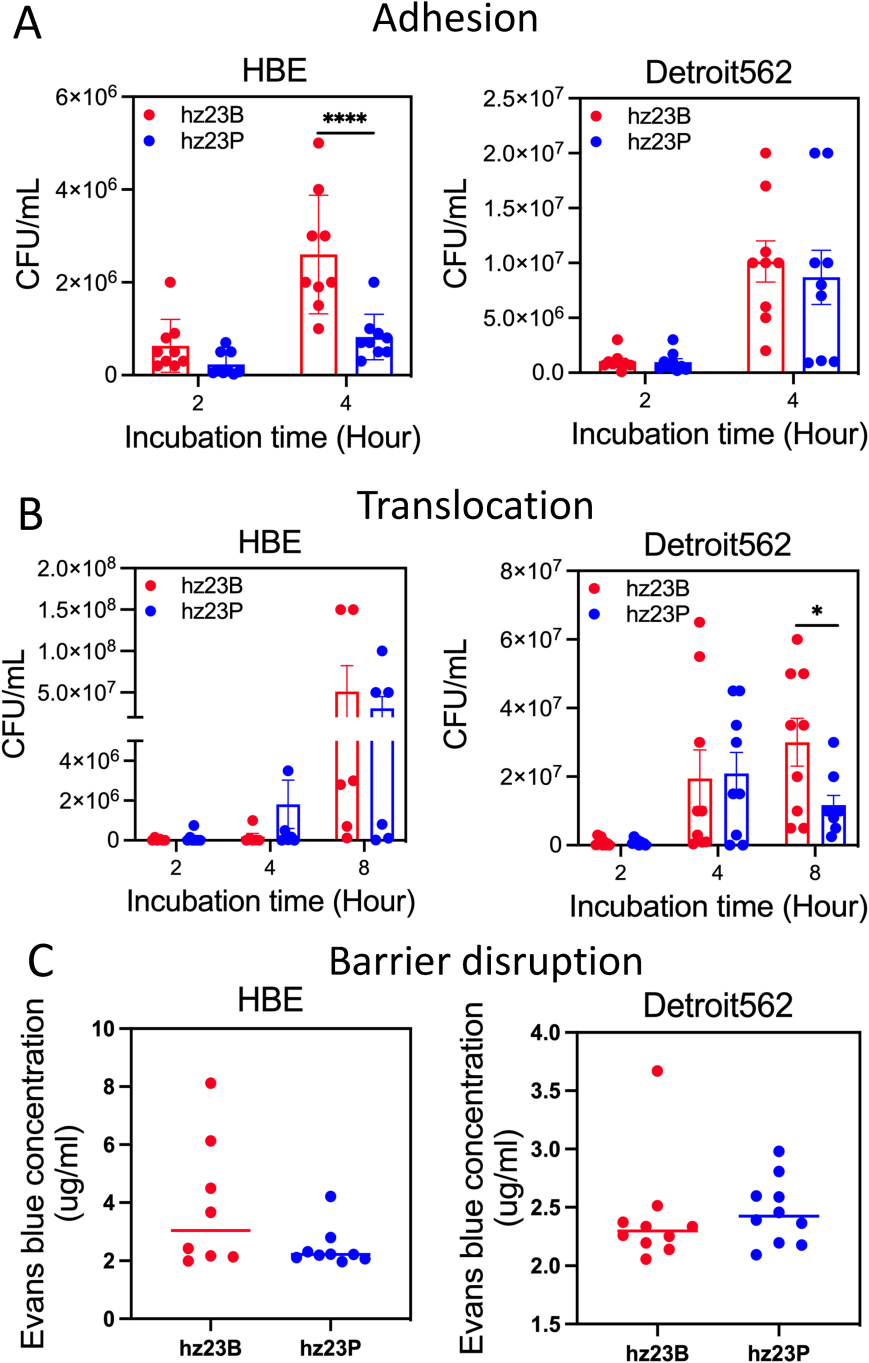
Adhesion and invasion test on human epithelial cells of hz23B and hz23P. The first panel shows the adhesion test results from both strains on lung cells HBE and human nasopharyngeal cells Detroit562 **(A)**. The second panel shows the translocation test results from both strains of isolates on lung cells HBE and human nasopharyngeal cells Detroit562 **(B)**. The third panel shows the barrier destruction ability of both strains on lung cells HBE and human nasopharyngeal cells Detroit562 **(C)**. * and **** represent a significant difference with a *p* < 0.05, *p* < 0.0001, respectively.

To further understand why hz23P, instead of hz23B, caused purulent pericarditis, we conducted a series of whole-genome analyses by including all pneumococcal serotype 14 strains (n=63) in our lab. As shown in the inner cycle of **Figure 2**, the phylogenetic analysis indicates the serotype 14 strains are relatively concentrated in genetic background, with most strains belonging to clone complex (CC) 876, including sequence type ST876 and four newly assigned STs: 19436, 19504, 19428, and 19519. Both hz23B and hz23P are ST876 strains, and we have only one pneumococcal strain isolated from pericarditis fluid, which is hz23P. The detection of virulent factors by blasting all serotype 14 strains against VFDB indicated a similar pattern in all tested strains, in which hz23B and hz23P carried the same virulent factors completely (**Supplementary Figure 1**). However, as displayed in **Figure 2**, mutation prediction analysis between hz23B and hz23P showed interesting results that two point mutations occurred in hz23P: W192L in CiaH and E21A in FabT. By analyzing the two genes encoding CiaH and FabT in all 14 serotype strains and the TIGR4 strain, we found that these two point mutations only appeared in strain hz23P (**Figure 3**; **Supplementary Figure 2**).

### Protein structure modeling

We further conducted protein structure modeling on CiaH and FabT to understand how these two mutations would affect the function of the proteins. As depicted in **Figure 2**. BC, sensor kinase CiaH of *S. pneumoniae* strain hz23P mutated W192L from strain hz23B, which is the homodimeric domain of signal transducing histidine kinase according to InterPro annotation. Thus, additional indole rings from mutation may cause tighter binding of homodimers, thereby changing the necessity for dimerization of the functional sensor kinase. The fatty acid synthesis gene repressor FabT of *S. pneumoniae* strain hz23P was mutated to E21A from strain hz23B, which is adjacent to an asymmetric homodimeric interface. The mutated residue of the cyan subunit loses an aceticoceptor, breaking two aromatic hydrogen bonds with histidine residues, resulting in a looser and unstable homodimer. The wild type of FabT dimer weakly binds to DNA (Zuo et al., 2019), while the mutation decreases its DNA-binding affinity further and renders its function nearly ineffective.

### Decreased virulence in pericarditis pneumococcal strain

Since we found the mutations in two important proteins in hz23P when compared with hz23B and all other serotype 14 pneumococcal strains, assessing their virulence, especially for their ability to adhere, permeability, and translocation on human cells, is necessary. A decreased virulence was observed for hz23P in human nasopharyngeal and bronchopulmonary epithelial cells. For instance, compared to hz23B, hz23P presented a similar adhesion ability when cultured with Detroit562 cells but had significantly (*p* < 0.0001) lower HBE cell adhesion at 4 hours (**Figure 3A**). In the translocation test (**Figure 3B**), we found both hz23B and hz23P had a similar ability to get through the nasopharyngeal epithelial barrier at the time point of 4 hours, but strain hz23B showed a stronger ability to pass Detroit562 cells at 8 hours. No translocation difference was observed for hz23B and hz23P when cultured with HBE cells. Lastly, the cell monolayer integrity status was the same when cultured for 8 hours with hz23B and hz23P for nasopharyngeal and bronchopulmonary epithelial cells (**Figure 3C**).

### Survival advantages of pericarditis pneumococcus in anaerobic and low-nutrient medium

**Figure 4:**
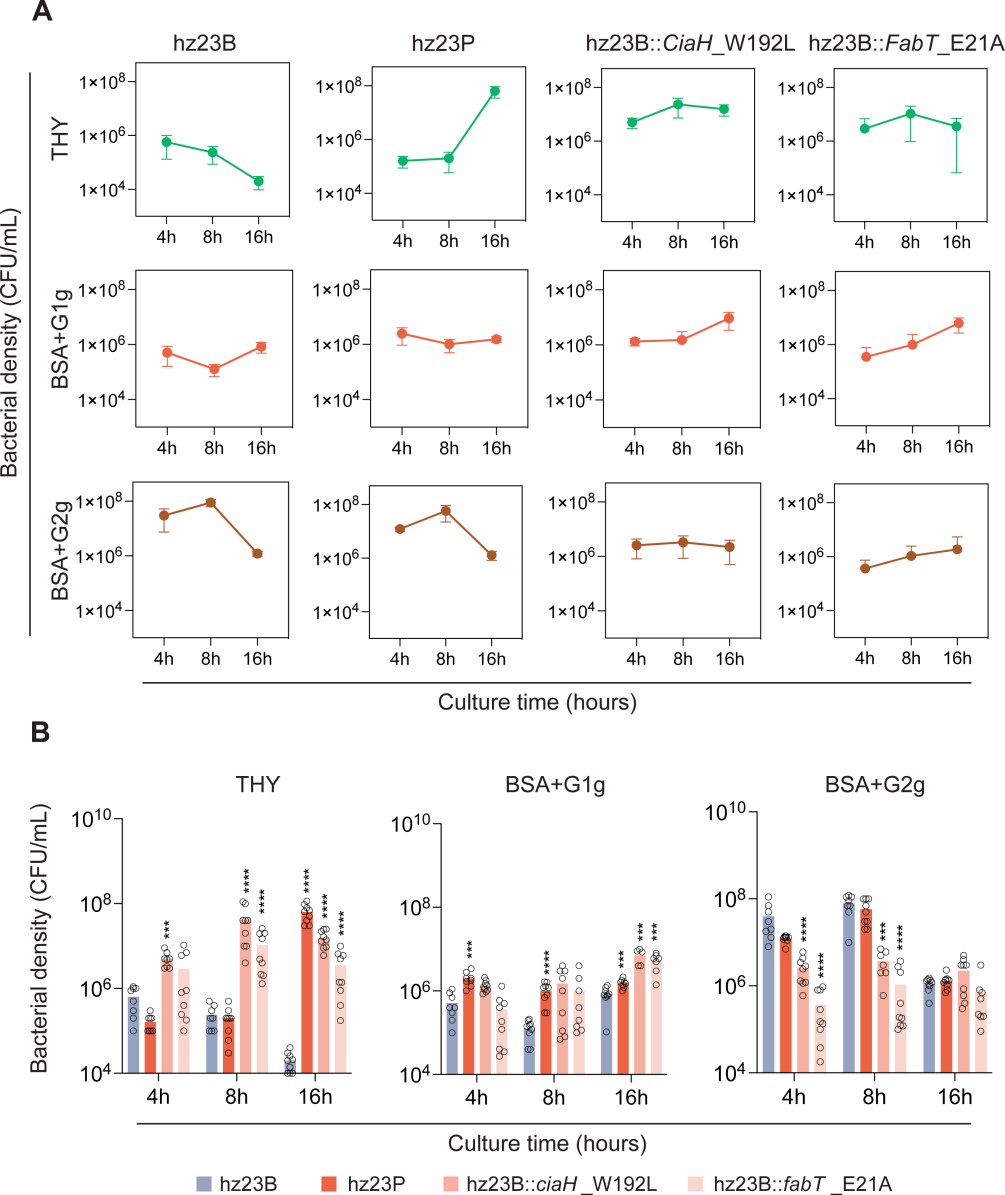
Survival test under an anaerobic environment. Bacterial counts of four tested strains cultured in rich-nutrition medium THY **(A)**, low-nutrition medium BSA+G1g **(B)**, and high-glucose medium BSA+G2g **(C)** for 4, 8, and 16 hours. hz23P exhibits a unique growth advantage under low-nutrient (BSA+G1g) and anaerobic conditions. Single-point mutants (hz23B::*ciaH*_W192L, hz23B::*fabT*_E21A) display partial or delayed phenotypes, suggesting synergistic interaction of the double mutation. **** and *** represent significant differences with *p* < 0.0001 and < 0.001, respectively.

To compare the survival difference between hz23B, hz23P, hz23B::*ciaH*_W192L, and hz23B::*fabT*_E21A, we conducted viable cell counting under an anaerobic environment in different culture media. As shown in **Figure 4**, as shown in **Figure 4**, in THY medium, hz23B and hz23P exhibited completely distinct growth patterns: hz23B failed to proliferate well under anaerobic conditions, whereas hz23P demonstrated a marked growth advantage at 16 hours. Meanwhile, neither hz23B::*ciaH*_W192L nor hz23B::*fabT*_E21A achieved the same level of growth advantage as hz23P at any time point, although both outperformed hz23B. This growth advantage of hz23P was not observed at 4 or 8 hours in THY medium. However, in BSA+G1g medium, hz23P showed a significant growth advantage over hz23B starting from 4 hours, while the two single-point mutants exhibited a growth advantage only at 16 hours. When the culture medium was replaced with BSA+G2g, neither hz23P nor the two single-point mutants of hz23B retained a survival advantage. In fact, both hz23B::*ciaH*_W192L and hz23B::*fabT*_E21A displayed a pronounced growth disadvantage.

### Global distribution of resistance and pericarditis-related mutations

**Figure 5:**
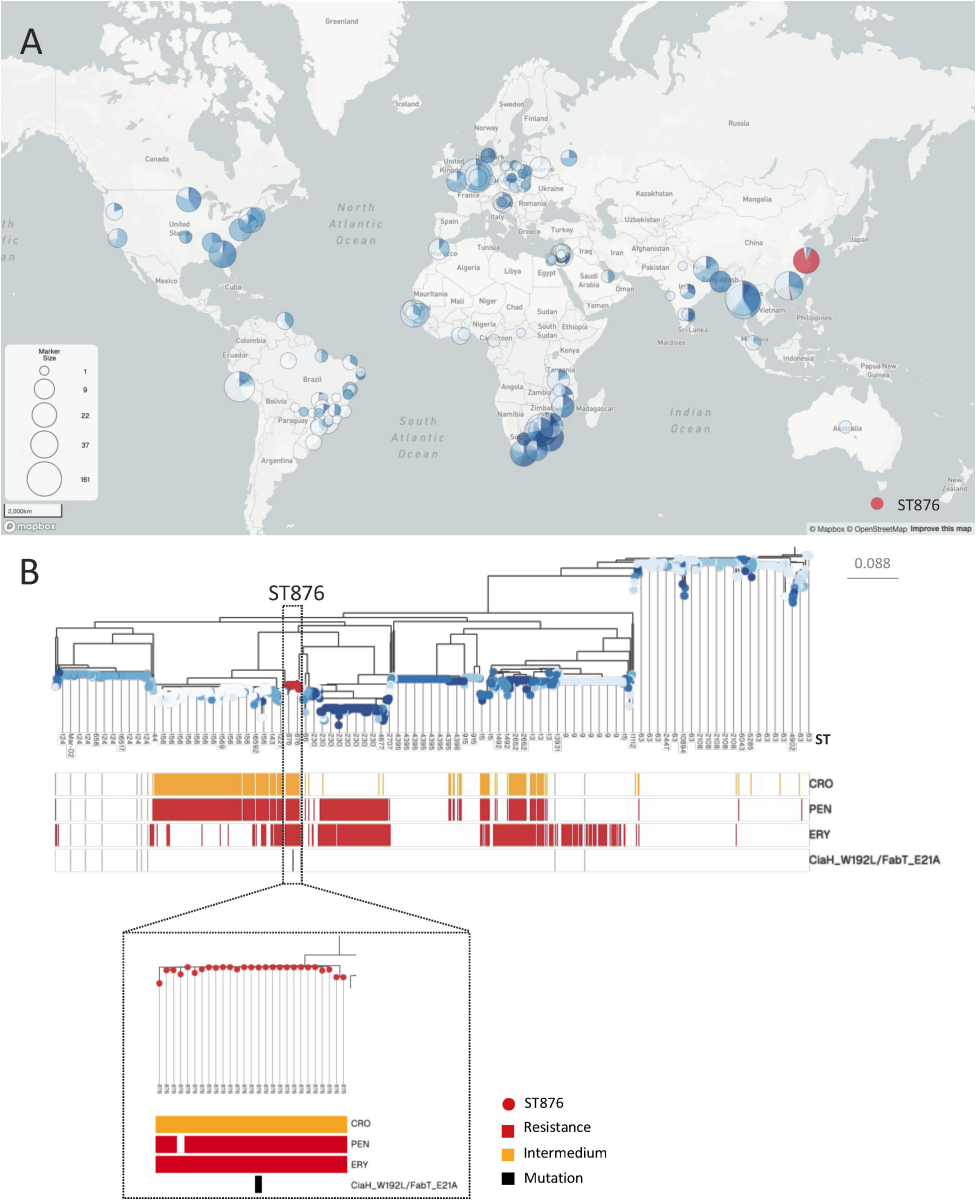
Global distribution of resistance and pericarditis-related mutations in serotype 14 strains. **(A)** The world map marked with the distribution of all sequence types (STs) of serotype 14 strains. The size of the circle indicates the number of isolates. The gradient blue shows the global distribution of different STs other than ST876, and red represents the occurrence of ST876; **(B)** The phylogenetic tree of all 1429 serotype 14 strains annotated with STs, CRO, PEN, and ERY non-susceptible (yellow) and resistant (red), CiaH_W192L/FabT_E21A (black) for each strain.

To further illustrate the distribution of resistance of key anti-pneumococcal agents and to identify and pericarditis-related mutations within serotype 14 strains, we analyzed 1429 global pneumococcal genomes. As shown in **Figure 5**, the ST876 strains are only prevalent in China and display a multidrug-resistant profile: non-susceptibility to CRO, resistance to PEN, and ERY. Moreover, the newly identified CiaH and FabT mutations are found exclusively in this ST876 lineage.

## Discussion

This study provides the first evidence of the in-host evolution of purulent pericarditis serotype 14 *S. pneumoniae*. Moreover, pneumococcal serotype 14 is covered by PCV13, which is on the market in China. Our findings again emphasized the virulence of ST876 serotype 14 pneumococcus and the importance of its immunization. Striking phenotypic differences were observed between the pericarditis isolate hz23P and its ancestral strain hz23B under various culture conditions, highlighting the adaptive significance of the identified mutations in *ciaH* and *fabT*. The adaptive phenotype of hz23P is not attributable to either mutation alone, but rather to a synergistic interaction between them and is nutrient-dependent. The double mutation enables a more rapid and robust adaptive response to nutrient limitation, potentially through metabolic reprogramming that optimizes energy utilization under stress. Further, both single-point mutants exhibited a significant growth disadvantage under high glucose conditions, suggesting that each mutation imposes a fitness cost when glucose is abundant. This cost may be mitigated by the compensatory effect of the double mutation in hz23P, further supporting a model of synergistic adaptation. These results demonstrate that the adaptive success of hz23P in nutrient-limited and anaerobic environments arises from the combined effect of mutations in ciaH and fabT, which together remodel central metabolism in a manner that is not simply additive.

It was reported that neutral evolution within the host was rapid and adaptive during colonization, while the genic evolution occurred particularly in antibiotic resistance, immune evasion, and epithelial adhesion genes (Chaguza et al., 2020). We detected two point mutations that caused amino acid differences in the proteins CiaH and FabT when the pneumococcus invaded the heart from the bloodstream. These mutations solely accrued in pericarditis effusion isolate hz23P compared to all other *S. pneumoniae*, indicating their important role during heart infection. The two-component system CiaRH in *S. pneumoniae* is responsible for competence development, antibiotic resistance, stress tolerance, and carbohydrate utilization (He et al., 2021; Pettersen et al., 2024). The protein CiaH is a histidine kinase sensor closely linked to a response regulator (CiaR) embedded in the bacterial cell membrane; a part of the protein is outside the cell, responding to environmental stimuli (He et al., 2021). Mutations in the transmembrane region of CiaH_A203V were reported to lead to increased antibiotic resistance (Guenzi et al., 1994; Giammarinaro et al., 1999; Müller et al., 2011). CiaH_W192L mutation in the current study is also in its transmembrane region, but we did not observe any antimicrobial resistance difference compared with its wild-type strain hz23B. However, we observed a significant growth superiority of hz23P in a low carbohydrate environment (BSA+G1g and 16 hours in THY) compared to hz23B. Mutations in the transmembrane part of CiaH may alter its kinase activity and ability to sense environmental signals, affecting the phosphorylation state of CiaR and the expression of downstream genes, thereby altering the carbon source utilization efficiency of *S. pneumoniae* (Mascher et al., 2006; Marx et al., 2014). According to our findings, the mutation in ciaH provides a growth advantage under conditions with low carbon sources. This may be related to the increased dependence of CiaR on acetyl phosphate for carbon source utilization. However, as this mechanistic hypothesis is derived from in silico predictions, it will require direct experimental validation in future studies.

Besides increasing resource utilization efficiency, reducing energy-consuming activities is crucial for bacteria to survive in a stressed environment. Fatty acid synthesis (FAS) is an energy-consuming fundamental metabolism pathway in *S. pneumoniae*, which is globally regulated at the transcriptional level by a MarR family repressor known as FabT (Zuo et al., 2019; Lambert et al., 2022). A 2.2 Å crystal structure of FabT in complex with a 23-bp DNA was reported previously (Zuo et al., 2019). The mutated residue of the cyan subunit results in a looser and unstable homodimer, which has been demonstrated to impair DNA-binding affinity (Alekshun et al., 2000; Deochand and Grove, 2017). Inactivation of the *fabT* gene in the *S. pneumoniae* D39 strain resulted in a colony phase variation with decreased capsule synthesis, adhesion, and invasion (Zhang et al., 2021). It is consistent with our findings that hz23P carries a mutation of FabT_E21A and presents an impaired adhesion and invasion ability. Through selection experiments on transposon-mutagenized *E. coli* libraries, researchers discovered that even when nutrients are extremely limited, significant adaptation can occur via loss-of-function mutations (Hottes et al., 2013). These mutations adjust cellular metabolism without enhancing enzymatic or sensory functions, which reduces energy waste and allows bacteria to focus on key survival and growth processes. Future studies, such as transcriptomic profiling under contrasting nutrient conditions, metabolic flux analysis, and enzymatic activity assays, will be essential to fully elucidate how these mutations fine-tune central metabolism in response to nutrient availability.

The global illustration reveals that ST876 serotype 14 behaves as a geographically confined yet high-risk clone. Its exclusive circulation within China might reflect regional vaccine pressure: PCV13 covers serotype 14, but the vaccine evasion of ST876 was reported (Lan et al., 2023), or an undefined fitness advantage under selective antimicrobial use in Chinese hospitals. The clone has accumulated characteristics of non-susceptibility to ceftriaxone, penicillin resistance, and erythromycin resistance, which effectively eliminates the three most commonly prescribed first-line agents for severe pneumococcal disease. More concerning is the co-occurrence of the CiaH_W192L and FabT_E21A mutations solely within ST876. CiaH modulates the CiaRH two-component system linked to *β*-lactam tolerance, while FabT is a master repressor of fatty acid biosynthesis genes, often in stress response. The coexistence of mutations in key metabolic and stress response genes suggests that this clone has undergone adaptive evolution. Like other highly virulent and resistant bacterial lineages, such convergent evolution may drive its dissemination (Zhu et al., 2020; Zhu et al., 2023; Zhu et al., 2024). The combined advantages for invasion and survival, amplified by antibiotic resistance, would promote this clone beyond sporadic outbreaks to international dissemination.

This study has several limitations. First, our findings are based on paired isolates from a single patient; validation in a larger cohort is needed to confirm the generalizability of the observed adaptive mutations. Second, although we successfully constructed single-point mutants of *ciaH* and *fabT*, we were unable to generate a double-point mutant in the hz23B background. This may reflect the challenges of genetic manipulation in clinical strains, and the growth defects of single mutants under high-glucose conditions suggest that the double mutation could impose a fitness cost. Future studies employing advanced gene-editing tools (e.g., CRISPR-Cpf1) to reconstruct the ciaH_W192L and fabT_E21A mutations in a standard laboratory strain background (e.g., D39 or TIGR4), or complementation studies in a successfully constructed double mutant, are required to definitively establish their precise roles in metabolic adaptation and virulence attenuation. Third, the *in silico* modeling offers a mechanistic hypothesis, but experimental validation is required to establish causality. Finally, while our findings implicate nutrient-dependent metabolic reprogramming involving CiaH and FabT, the precise molecular mechanisms remain to be elucidated.

## Conclusion

Our study emphasizes the importance of prompt diagnosis and treatment of purulent pericarditis caused by *S. pneumoniae*, highlighting the need for ongoing surveillance of antibiotic resistance. After invading the host pericardium, *S. pneumoniae* shifted its carbohydrate utilization to an energy-saving model by altering the transmembrane structure of CiaH, also transitioning into a low-virulence state to conserve energy through a loss-of-function mutation in its Fatty acid synthesis pathway, allowing it to survive in the low-oxygen, nutrient-poor pericardial environment. Moreover, ST 876 serotype 14 is a hypervirulent, multidrug-resistant pneumococcal clone that is ready to breach geographical boundaries and seed a worldwide emergence, demanding urgent global attention.

## Data Availability

The datasets presented in this study can be found in online repositories. The names of the repository/repositories and accession number(s) can be found in the article/**Supplementary Material**.
