# Supplementary material for: Emergence of multidrug-resistant ST876 serotype 14 Streptococcus pneumoniae with spontaneous mutations driving pathogenesis: genomic and clinical insights from pneumococcal pericarditis and global screening
Source: Front Cell Infect Microbiol. 2026 Mar 24;16:1723478. doi: 10.3389/fcimb.2026.1723478 (PMC13053516; doi:10.3389/fcimb.2026.1723478)
Supplement: Supplementary file 1 [file DataSheet1.docx]

**Supplementary materials**

**
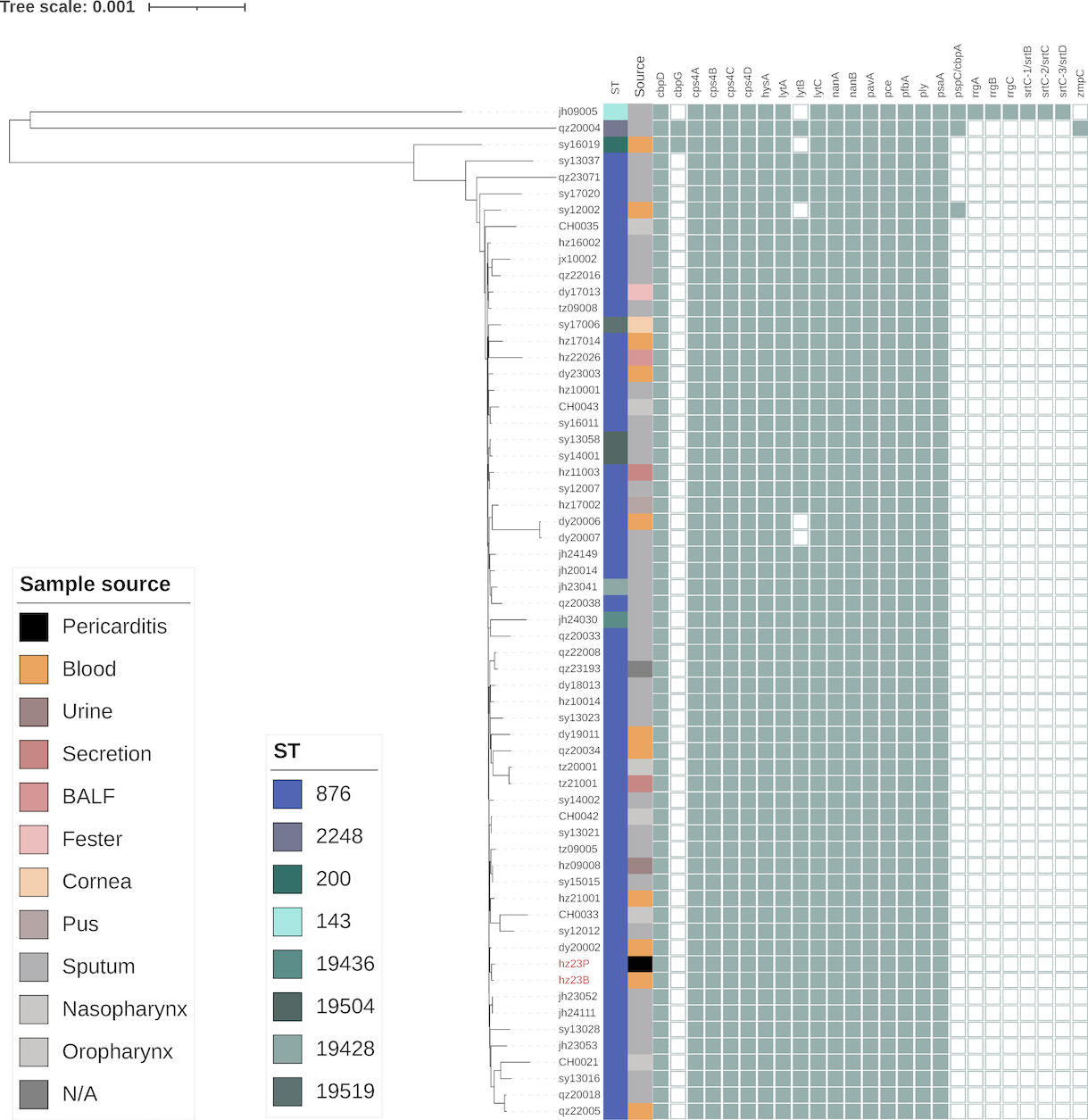
**

**Supplementary Figure 1. Virulence factor detection of all serotype 14 *S. pneumoniae***. The phylogenetic tree was annotated by the sequence type (ST) of each tested pneumococcal strain. ST876 and four newly assigned STs: 19436, 19504, 19428, and 19519 were clustered in one clone complex. The sample source was also marked afterwards. The binary dataset indicates the virulence factors screen result for each strain, where green shows the positive detection.


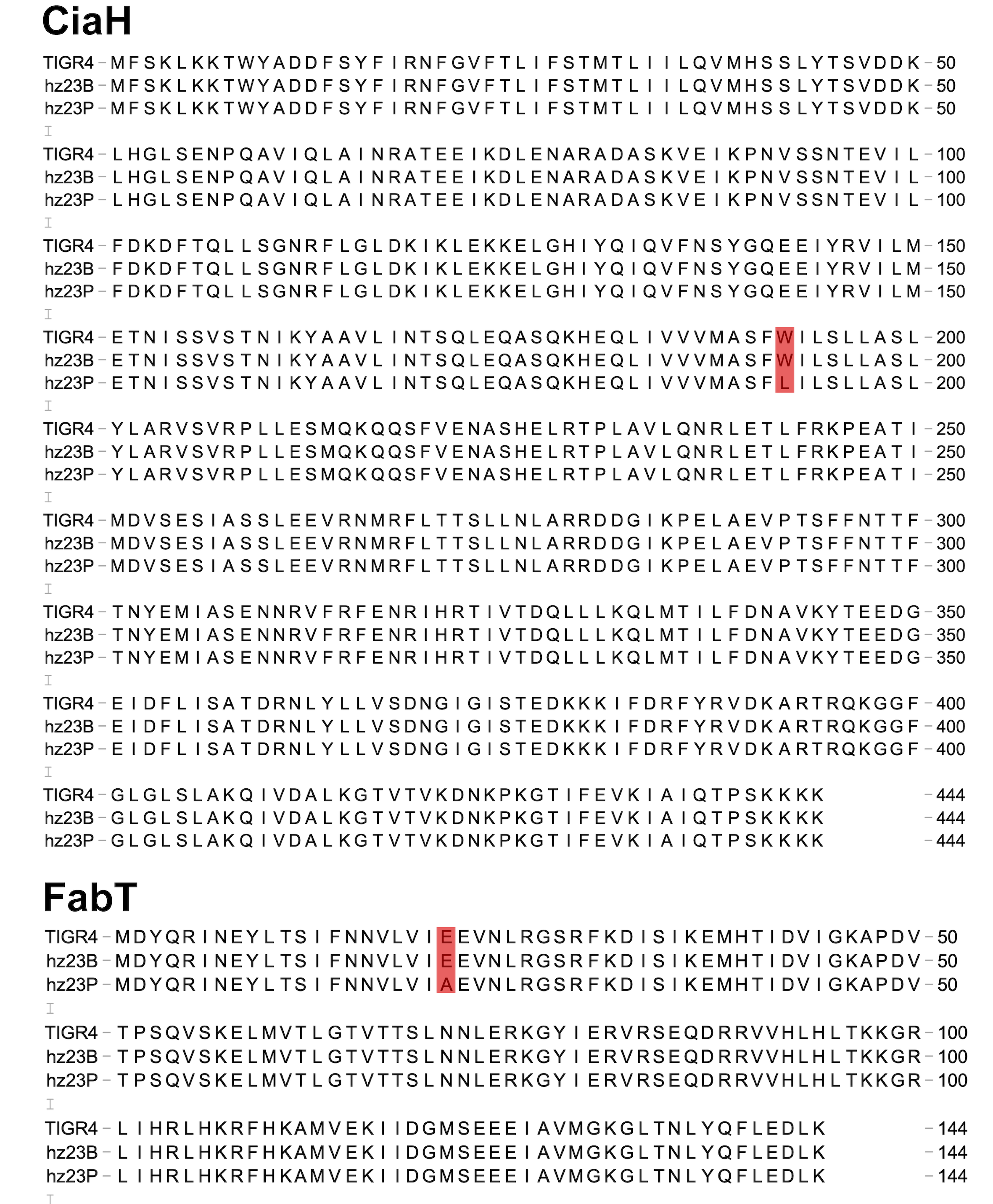


**Supplementary Figure 2. Protein sequence mapping of CiaH and FabT in hz23B, hz23P, and TIGR4.** The red color marked the mutation site for each protein.
